# Measurements of Nuclear Magnetic Shielding in Molecules

**DOI:** 10.3390/molecules29112617

**Published:** 2024-06-02

**Authors:** Karol Jackowski, Marcin Wilczek

**Affiliations:** Laboratory of NMR Spectroscopy, Faculty of Chemistry, University of Warsaw, Pasteura 1, 02-093 Warsaw, Poland; wilczek@chem.uw.edu.pl

**Keywords:** NMR spectroscopy, magnetic shielding, chemical shifts, nuclear magnetic moments, measurements of shielding

## Abstract

The origin of nuclear magnetic shielding in diamagnetic molecules is discussed, pointing out various contributions to the shielding from electrons and the effects of intra- and intermolecular interactions. In NMR practice, chemical shifts are determined first as the measure of shielding in observed samples. The descriptions of shielding and chemical shifts are not fully consistent. Gas phase studies permit the withdrawal of intermolecular contributions from shielding and obtaining the magnetic shielding data in isolated molecules. The shielding determination in molecules is possible using at least three methods delivering the reference shielding standards for selected nuclei. The known shielding of one magnetic nucleus can be transferred to other nuclei if the appropriate nuclear magnetic moments are available with satisfactory accuracy. It is possible to determine the nuclear magnetic dipole moments using the most advanced ab initio shielding calculations jointly with the NMR frequencies measurements for small-sized isolated molecules. Helium-3 gas is postulated as all the molecules’ primary and universal reference standard of shielding. It can be easily applied using common deuterium lock solvents as the secondary reference standards. The measurements of absolute shielding are available for everyone with the use of standard NMR spectrometers.

## 1. Introduction

Electrons always shield atomic nuclei in molecules from the influence of an external magnetic field. This physical phenomenon is described by the difference between the induction of applied field *B_0_* and its value *B_eff_* experienced by the nucleus and for isotropic species is expressed as follows:

(1) $$B_{eff}=\left(1-\sigma \right)B_{0}$$

where *σ* is the shielding parameter dependent on the total electronic structure of the observed molecular system, the above form of Equation (1) means *σ =* 1/3 *(σ_xx_ + σ_yy_ + σ_zz_)* because in a general case, shielding is a second-rank tensor and the induction of magnetic field is represented by its vectors. Equation (1) is sufficient for spherical systems like atoms or molecules in the gaseous and liquid states where molecular reorientation is not hindered. For atoms, the shielding is just described by the Lamb [1] equation [2]. The shielding theory for molecules is a bit more complex and was first formulated by Ramsey for diatomic molecules, especially for a hydrogen molecule [3,4]. In 1954, Saika and Slichter [5] noted that the magnetic shielding of a nucleus *σ_i_* can be presented as the sum of three different components

(2) $$\sigma_{i}=\sigma_{i}^{d}+\sigma_{i}^{p}+\sum_{i\neq j}\sigma_{j}$$

In this equation, *σ_i_^d^* and *σ_i_^p^* are the local diamagnetic and paramagnetic parts of shielding when the last term is responsible for all the modifications of *σ_i_* arising from intra- and intermolecular effects. A more detailed description of the shielding in diamagnetic molecules was provided by Pople [6] and other researchers [7,8]. They explained the first two partial terms in Equation (2) as follows [9]:

(3) $$\sigma_{i}^{d}=\left(\frac{\mu_{0}}{4\pi }\right)\left(\frac{e^{2}}{3m_{e}}\right)\left\langle 0\right.|\sum_{n}\frac{1}{r_{n}}|\left.0\right\rangle$$

(4) $$\sigma_{i}^{p}=-\left(\frac{\mu_{0}}{4\pi }\right)\left(\frac{e^{2}}{3m_{e}}\right)\sum_{k\neq 0}\frac{1}{E_{k}-E_{0}}\left(\left\langle 0\right.|\sum_{n}L_{n}|\left.k\right\rangle \left\langle k\right.|\sum_{n}\frac{L_{n}}{r_{n}^{3}}|\left.0\right\rangle \right)$$

**Figure 1:**
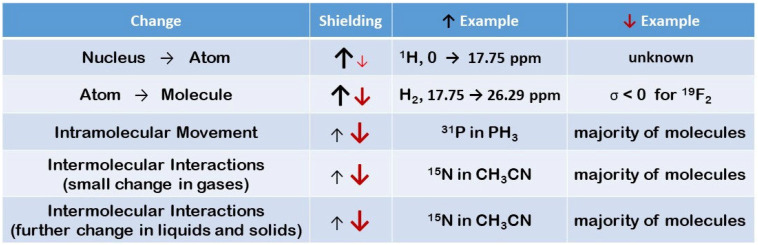
Selected examples from multinuclear NMR spectroscopy qualitatively illustrate the origin and further modifications of nuclear magnetic shielding in diamagnetic molecules. Black arrows mean the increase in shielding, and red arrows represent the deshielding effects.

Equations (3) and (4) contain constants: *e*—electron charge, *m_e_*—electron mass, and *µ_0_*_—_free space permeability. Then, in the bra-ket notation, there are the described wave functions of the ground state of electrons (0) and all the excited states (k) with their appropriate energies *E_0_* and *E_k_*. ***L*** and ***r*** are the vectors that represent the orbital angular momentum and the distance from an arbitrary origin for the nth electron, respectively. The important features of Equations (3) and (4) arise from the different signs of these two components to the total magnetic shielding: *σ_i_^d^* is positive (shielding effect) while *σ_i_^p^* is always negative (deshielding effect). It means that the total shielding effects for diamagnetic molecules can be positive (*σ_i_* > 0) or negative (*σ_i_* < 0), assuming $\sum_{i\neq j}\sigma_{j}=0$ in Equation (2). The last term of Equation (2) is responsible for intra- and intermolecular effects that can be separately measured in the gas phase as described by Jameson [10,11]. All the possible components of Equation (2) are presented in Figure 1 using selected examples from multinuclear NMR spectra.

As seen in Figure 1, the magnetic properties of atoms and molecules are the function of their electronic structures. Atoms are usually more shielded, having more electrons, but atomic electronegativity is also an important feature. Molecules have a more complex distribution of electrons around atomic nuclei, and all the terms of Equation (2) must be remembered. The total shielding can be negative for some atomic nuclei; the F_2_ molecule is a spectacular example of such a case but is not unique. There are many other examples of the total deshielding effects for diamagnetic molecules, especially observed in ^15^N, ^17^O, and ^19^F NMR spectra. However, more electrons in the neighborhood usually lead to an increase in nuclear magnetic shielding in diamagnetic molecules. Molecular vibrations and rotation cause further changes in shielding because the interatomic distance is enlarged. It results in deshielding effects in the majority of molecules, but some exceptions from the above rule are also known, cf. PH_3_ molecule shown in Figure 1. All intermolecular interactions also modify nuclear shielding in diamagnetic molecules; usually, the decrease of shielding is observed, but the reverse effects occur for selected atoms containing lone pairs of electrons. Figure 1 illustrates such a case for a ^15^N nucleus in acetonitrile.

Gas phase studies are crucial for the separation of molecular shielding parameters from all the large intermolecular contributions present in macroscopic samples. The same effects are still present in the gaseous samples, but they are much smaller and can be eliminated by the extrapolation of shielding measurements to the zero-density limit [10,11]. It gives the measurement of shielding equivalent to isolated molecules denoted as *σ*_0_. Our review is mostly focused on the shielding in isolated molecules because only such experimental results can be used to connect with the most advanced calculations of the same parameters. Let us add that the description of nuclear magnetic shielding given by Equations (2)–(4) is very useful in the qualitative understanding of shielding in diamagnetic molecules, but such an approximation is old-fashioned and is not applicable to modern quantum chemical calculations. The present state-of-the-art calculations of shielding [12,13,14,15,16,17,18] deliver such excellent results that they can often be treated as the source of the best data even in experimental NMR work, as shown in the recent comparison of experimental and calculated NMR parameters in CH_4-n_F_n_ molecules [19]. It is nothing unusual, but verifying all available calculated results by experiment is also welcomed because many different approximate quantum chemistry methods are widely applied to shielding calculations.

In molecules, atomic nuclei are always surrounded by electrons, and so far, there is no possibility for the straightforward measurement of the molecular shielding, *σ_i_*. NMR spectroscopy offers only a reading of the shielding difference between two macroscopic shielded objects, known as a chemical shift. This parameter is extremely helpful for the qualitative analysis of chemical compounds but contains rather limited information on shielding. Nevertheless, chemical shifts are also exploited to determine nuclear magnetic shielding as the first step when the absolute shielding of at least one reference standard is known satisfactorily. The problem of absolute shielding is not easy because it usually requires additional information from other than NMR experiments or quantum chemical calculations, and, often, it must be solved individually for each kind of magnetic nucleus. At least three methods are applied to the above investigations and are discussed in the present review. All the experimental attempts of shielding determination are equally important and precious because they allow for the reliable verification of the determined σ_0_ data in molecules.

## 2. NMR Chemical Shifts

NMR spectra are most applied in the qualitative analyses of chemical liquid compounds, and the shielding parameters are observed as the NMR chemical shifts (*δ_i_*) measured relative to selected reference standards:

(5) $$\delta_{i}=\frac{\sigma_{ref}-\sigma_{i}}{1-\sigma_{ref}}\approx \sigma_{ref}-\sigma_{i}$$

where *σ_ref_* and *σ_i_* are the shielding values of reference and investigated compounds, respectively. This is an excellent method for analytical chemistry but requires strict standardization of chemical shift measurements, as described by Harris et al. [20,21].

It is accepted in the scientific literature that NMR chemical shifts were discovered by Proctor and Yu when separate ^14^N signals from NH_4_^+^ and NO_3_^-^ ions were observed at different resonance frequencies with a constant magnetic field [22]. The same issue of Physical Review also contains Dickinson’s publication [23], which describes the observation of ^19^F signals of fluorine compounds with various magnetic fields at the fixed resonance frequency. Both the research notes report the same finding of chemical shifts because the NMR experiment can be completed by either changing resonance frequencies with the stable magnetic field (*B*_0_) or having a constant electromagnetic frequency (*ν*_0_). One can use the variation of a frequency (or a magnetic field) as shown by Equations (6) and (7).

(6) $$\delta_{i}=\frac{\nu_{i}-\nu_{ref}}{\nu_{ref}}\;for\;B_{0}=const.$$

(7) $$\delta_{i}=\frac{B_{ref}-B_{i}}{B_{ref}}\;for\;\nu_{0}=const.$$

*δ_i_* is the parameter earlier defined by Equation (5), and the above equations only show how the NMR measurement should be performed. The indexes “i” and “ref” are for the observed and reference nuclei. Chemical shifts are usually in the range of 10^−6^_,_ and their values are always presented in “parts per million” (in ppm’s). In the first 25–30 years of NMR history, Equation (7) was mostly used for the determination of chemical shifts. Later, the superconducting magnets were introduced to standard NMR spectrometers, and Equation (6) became the formula recommended by IUPAC [20,21].

Let us note that Equations (6) and (7) have different orders of “i” and “ref” indexes in the formulas. It is true because the increase of radiofrequency (*B*_0_ = const.) is observed for less shielded nuclei while the magnetic field (*ν*_0_ = const.) and magnetic shielding are changing in the same direction. It arises from the basic description of an NMR experiment when the *hv* quantum of radio-frequency energy is absorbed by the single magnetic nucleus:

(8) $$h\nu =g_{X}\mu_{N}\left(1-\sigma \right)B$$

where *g_X_* is the *g*-factor of the observed nucleus (*g_X_* = *µ_X_/(I*_X_*µ_N_),* which describes its spin *I_X_*, magnetic moment *µ_X_*, and *µ_N_* is the nuclear magneton. Over one hundred stable atomic nuclei are magnetic and each kind of them has its magnetic moment and the appropriate *g_X_* value. Consequently, there are as many NMR spectroscopic methods as the number of magnetic nuclei. Some of them, e.g., ^1^H, ^13^C, ^15^N, ^17^O, ^19^F, and ^31^P NMR, are especially important in organic chemistry. The others are also intensively explored in experimental studies of chemistry and physics. Table 1 presents only the modest choice of magnetic nuclei and their NMR spectral parameters used for the discussed methods of shielding measurements.

As seen in Table 1, the selected well-known nuclides have mostly a spin number *I_X_* equal to ½. Such nuclei are spherical and have no quadrupole electrical momentum. Their NMR signals are usually sharp and permit more precise measurements of chemical shifts. Nevertheless, Table 1 also includes oxygen-17 and deuterium because of their importance in NMR spectroscopy and the presentation of some problems discussed in this review. The next column of Table 1 gives the natural abundance of the magnetic nuclei. Usually, the natural abundance above one percent guarantees good NMR spectra if a modern FT spectrometer is applied. A sample containing a much lower percentage of magnetic nuclei cannot be easily observed at the natural abundance, especially in the gas phase. The magnetic moments and *g_X_* factors describe the magnitude of nuclear magnetic properties; their improved values are given in [24].

There is also one more interesting column in Table 1, which reveals the absolute resonance frequencies of selected reference standards if the various NMR experiments are performed when the external magnetic field (*B*_0_) is fixed and giving a ^1^H NMR signal of 1% TMS in CDCl_3_ precisely at *Ξ_H_* = 100.000000 MHz [20]. This idea of the absolute chemical shift comes from early double resonance experiments [33,34] and is very interesting because it unifies the important NMR parameter (chemical shift) for all magnetic nuclei. Unfortunately, it requires the double resonance method and involves many other experimental problems that the concept of *Ξ_X_* has never been popular in everyday use of NMR practice. Limiting the discussion only to proton spectra, we should write the following *Ξ_H_* values for isolated molecules of methane (100.0002847 MHz), ethane (100.0003593 MHz), and ethylene (100.0008017 MHz) based on existing *δ_i_* results [35]. It is not so bad when everything is limited only to ^1^H NMR experiments. However, too many digits present at every NMR measurement make such a description of experimental data impractical and unpopular in everyday NMR experimental work. It is also important that for all nuclei other than protons, the *Ξ_X_* measurement requires the simultaneous observation of ^1^H NMR experiment because from the definition *Ξ_H_* (1% TMS in CD_3_Cl) must be equal to 100.000000 MHz in every case. It cannot be achieved using a standard NMR spectrometer.

## 3. Nuclear Magnetic Shielding in Molecules

### 3.1. General Insight

**Figure 2:**
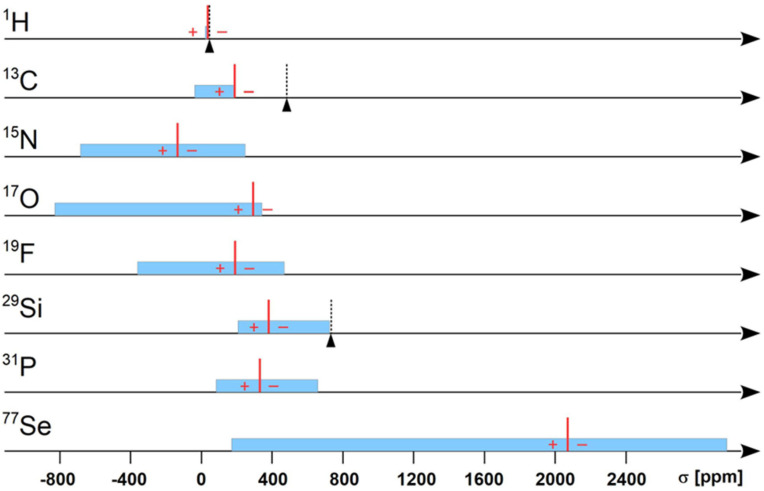
On the scale of nuclear magnetic shielding, the blue bars represent the range of chemical shifts for selected nuclei [43]. The values of chemical shifts can be positive (+) or negative (−) depending on the position of the reference standard (I). Special shielding effects are also highlighted by black triangles for three compounds containing iodide atoms: HI in ^1^H, CI_4_ in ^13^C, and SiI_4_ in ^29^Si NMR [35,44,45,46,47].

The range of chemical shifts depends on the electronic structure of atoms in molecules and is different for each kind of magnetic nuclei. Figure 2 illustrates the approximate area of chemical shifts on the scale of the nuclear magnetic shielding for the most popular nuclei and the positions of the recommended reference standards. The diagram refers to Table 1 but lacks two nuclei: ^2^H and ^3^He. The range of ^2^H NMR chemical shifts is practically the same as for ^1^H NMR if the minimal ^2^H/^1^H isotope effects in shielding are neglected [36]. Helium-3 is not present here because it does not form any chemical compound. There is only a special case of ^3^He NMR chemical shifts when helium atoms are encapsulated in fullerenes; then, the helium-3 shifts are −6.4 ppm for ^3^He@C_60_ and −27.9 ppm for ^3^He@C_70_ systems relative to pure gas ^3^He [37,38]. Theoretical models of helium-3 encapsulated in more complex carbon nanostructures predict even larger effects in ^3^He shielding [39,40], and all recent results on helium-3 NMR were overviewed by Kupka [41] and Krivdin [42].

Figure 2 has been prepared mostly using the available data on chemical shifts given by Bruker Almanac 2010 [43] and the shielding parameters of reference standards shown in Table 1. The blue bars approximately cover the ranges of ^1^H, ^13^C, ^15^N, ^17^O, ^19^F, ^29^Si, ^31^P, and ^77^Se magnetic shielding for diamagnetic chemical compounds. There are also marked three selected special points of shielding: σ_H_(HI) = 43.92 ppm (cf. *δ*_H_ = −10.44 ppm for HI in ^1^H NMR [35,44]), σ_C_(CI_4_) = 478.7 ppm (*δ*_C_ = −292.3 ppm for ^13^CI_4_ [45,46]), and σ_Si_(SiI_4_) = 730.7 ppm (*δ*_Si_ = −351.7 ppm for ^29^SiI_4_ [47]). All the latter chemical shifts are measured relative to TMS and illustrate the unusual increase of shielding due to the presence of so-called “heavy atoms”, an iodide in this case [48,49]. The effect is especially enormous for ^13^C NMR spectra where the signal of carbon tetraiodide (CI_4_) is far away from the normal range of carbon-13 chemical shifts. It is so strong that even solvent molecules containing halogen atoms produce extremely large intermolecular shifts in ^13^C NMR spectra [50,51].

Figure 2 shows only a modest representation of over 100 stable magnetic nuclei, which can be observed by the NMR method, and it proves how different the features of shielding for various magnetic nuclei are. First, there is a different range of shielding for each element. A shielding range of only several ppm for proton spectra and almost three thousand for ^77^Se, and even more for heavier nuclei like ^199^Hg or ^205^Pb. Second, the particular area of shielding belongs to its characteristic region. It is so different if we compare the scales of ^17^O and ^77^Se shielding, less positive for oxygen-17 and more positive for selenium-77. Third, the reference standard is usually different for each magnetic nucleus, with one significant exception. Tetramethylsilane (TMS) was introduced as the internal reference standard to ^1^H NMR by Tiers in 1958 [52] and later accepted also for the referencing of ^13^C and ^29^Si spectra. Multinuclear NMR experiments also varied in many other details, such as the natural abundance of each nucleus or the magnitude of its nuclear magnetic moment. As seen in Figure 2, the whole multinuclear NMR spectroscopy is well described and unified by one common parameter—nuclear magnetic shielding.

Therefore, NMR shielding is an important parameter of molecules and can be determined from chemical shifts using Equation (5) if the shielding of at least one small molecule (*σ_ref_*) for each magnetic nuclide is known with satisfactory accuracy. There is a fundamental question of how the *σ_ref_* reliable value for the given nucleus can be determined. It seems that the most direct method is available just from advanced quantum chemical calculations.

### 3.2. Theoretical Approach to Shielding

The shielding ab initio calculations usually start with a molecule’s computed equilibrium geometry. It requires large basis sets for the satisfactory description of electron correlations in small molecules. The common solution to this problem applies the gauge-included atomic orbitals (GIAO) approach [12,13]. Various approximate methods are used for the shielding parameter calculations (frequently named improperly as the shielding constants): from HF (Hartree-Fock) to FCI (Full Configuration Interaction), MCSCF (Multi-Configuration Self-Consistent-Field), CC (Coupled Claster) approximation and MP (Møller–Plesset) perturbation theory [17]. The reference NMR molecule is usually the smallest one, and for this purpose, we should look for the most advanced ab initio methods with the perturbative-dependent basis set. Electron correlation effects should be calculated at CCSD (Coupled Cluster Singlets and Doubles) or CCSD(T) (Coupled Cluster Singlets and Doubles with Perturbative Triple Corrections) levels of theory [14,15]. The modern state-of-the-art magnetic shielding calculations are fairly advanced at the non-relativistic level [53]. They include all the intra- and intermolecular contributions to shielding [54], as such effects are always present in NMR experiments. To obtain calculated results that are ready for comparison with the experiment, it is necessary to consider the strong dependence of shielding on molecular geometry. It can be achieved at the ZPV (Zero-Point Vibration), or even better, including temperature effects up to the value of experimental work. Finally, the relativistic effects in shielding should be considered, mainly responsible for the extensive shielding of heavy atoms. It requires new methods designed for the description of electron interactions in molecules (four-component Dirac-Coulomb-Breit) [55]. Relativistic calculations are more expensive than any non-relativistic methods, and less advanced descriptions of electrons are usually applied, like HF or density functional theory (DFT) approximation. The relativistic contributions to shielding are mainly responsible for the extensive shielding of ^1^H in HI, ^13^C in CI_4_, and ^29^Si in SiI_4_, as shown in Figure 2. It is the so-called HALA effect (heavy-atom effect on light atoms).

Intermolecular effects are difficult for theoretical treatment [17,54], and for this reason, it is better to avoid such effects by performing NMR experiments in the gas phase. Recently, a precise comparison of experimental and calculated shielding for small molecules CH_4-n_F_n_ has been presented [19]. Earlier, a similar comparison was performed for NF_3_, PF_3_, and AsF_3_ compounds, where the change of absolute ^19^F shielding in liquid CFCl_3_ to 197.07 ppm was suggested [56]. It is questionable and requires new experimental proof because the existing last measurement gave 190.0 ppm if a bulk susceptibility correction is excluded [57,58]. The recent comparison of ^19^F shielding in CH_4−n_F_n_ [19] rather confirms the old experimental result for liquid CFCl_3_.

The new methods of shielding calculations are powerful for small molecules, but let us compare the first approximation of proton shielding in the H_2_ molecule given by Ramsey in 1950 [3] (26.8 ppm) with the available calculated results: Sundholm and Gauss CCSD(T) value is 26.2983 ppm at T = 298 K [59] and Jaszuński et al. CCSD shielding is 26.2980 at T = 300 K [60]. Ramsey’s prediction of ^1^H shielding in H_2_ molecule is overestimated only by 1.9 percent. The above example of magnetic shielding in the hydrogen molecule also illustrates the importance of rovibrational effects in shielding [54] that were included in the new calculations and effectively diminished the final H_2_ result [59,60].

We are mostly interested in the nuclear magnetic shielding observed in small molecules, but quantum chemical calculations are also possible for larger molecular objects if some further approximations are applied. In such a case, the most popular method is DFT (Density-Functional Theory) [16], which can also be applied to studies of shielding at the four-component relativistic level [46]. Another approach is offered by the ONIOM method, in which the shielding calculations are performed with different approximation levels for the selected layers of electrons in a molecule [61]. It permits more precise calculations of shielding in larger molecules, simultaneously saving computer time. Other mixed methods of shielding calculations are applied for solids. Recently, it has been shown that a quantum mechanics/molecular mechanics (QM/MM) method can predict the solid-state NMR shielding for molecular crystals [62].

## 4. Determination of Absolute Shielding

### 4.1. The Ramsey–Flygare Method

Equations (2) and (3) reveal that the magnetic shielding in a molecule consists of the positive diamagnetic part (*σ_dia_*), which depends only on the ground electronic state of the molecule. This shielding part can be relatively easily calculated using modern quantum chemical methods. The second term of shielding described by Equation (4) is much more complex for calculations but is related to the microwave nuclear spin–rotation tensor (**C**), which is reduced to a coupling constant (*c_I_*) for linear molecules. It happens because, in such a case, the paramagnetic shielding part parallel to the bond axis (*σ_‖_^p^*) is equal to zero. Remains the perpendicular part of paramagnetic shielding (*σ_Ʇ_^p^*), which, as shown by Flygare [63,64], is related to the spin-rotation constant for a nucleus in a diatomic molecule as follows:

(9) $$\sigma_{⊥}^{p}=\frac{3}{2}\sigma^{p}=-\left(\frac{m_{p}}{2m_{e}g_{X}}\right)\left(\frac{c_{I}}{B}\right)-\frac{3}{2}\left(\frac{\mu_{0}}{4\pi }\right)\left(\frac{e^{2}}{3m_{e}}\right)\left(\frac{Z}{r}\right)$$

In the above equation, *m_p_* is the proton mass, *B* is the rotational constant of the molecule, *Z* is the atomic number of the neighbor atom in the molecule, and *r* is the internuclear distance. The rest of the parameters (*m_e_*, *µ_0_*, *e*, and *g_X_*) are the same as previously defined in Equations (3), (4), and (8), respectively. As seen in Equation (9), there is a link between the spin-rotation interaction of microwave spectroscopy and the absolute nuclear magnetic shielding observed in NMR [65]. However, the procedure of its use requires more additional work: first, the experimental spin-rotation constant must be refined from its vibrational corrections, and then the equilibrium value of shielding is obtained as shown in Equation (9), and finally, the rovibrational corrections should be added to shielding to obtain the *σ_ref_* value at the given temperature (usually 300 K). This relationship was exploited for the determination of absolute nuclear shielding in hydrogen fluoride (HF) delivering *σ_ref_*(^19^F) [57], in carbon monoxide molecules (^13^C^16^O and ^12^C^17^O) delivering the important *σ_ref_*(^13^C) [66,67,68] and *σ_ref_*(^17^O) values [69,70], respectively. The oxygen-17 case contains an interesting feature: it was initially based on the rotational constant of the ^12^C^17^O molecule obtained from the observation of the J = 0 ← 1 l transition in the rotational spectrum observed from interstellar space [71] until a more accurate measurement of the same rotational constant was available from the laboratory experiment in 2002 [72]. It is important to note that the Ramsey–Flygare method is not limited to linear and diatomic molecules [64]. It was possible to obtain the *σ_ref_*(^15^N) value for ammonia enriched in nitrogen-15 [73] and *σ_ref_*(^31^P) for PH_3_ [74].

### 4.2. Methods Based on ^1^H NMR Signal of Liquid Water and Shielding Transfers

**Figure 3:**
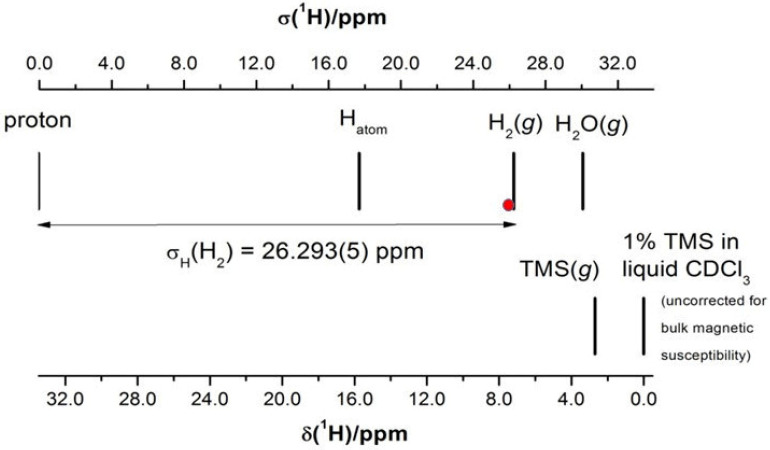
Alternative reference standards of shielding and chemical shifts for ^1^H NMR: the red dot (•) represents the shielding of liquid water in a spherical sample at 34.7 °C *σ_ref_*(H_2_O_liq.sph_) = 25.790(14) ppm [77], *σ*_0_(H_2_) = 26.293(5) ppm [35], *σ*_0_(H_2_O) = 30.102(8) ppm [29], and *σ*_0_(TMS) = 30.783(5) ppm [25]; *σ*(1% TMS in liq. CDCl_3_) = 33.480(5) ppm as shown in Table 1.

The second method of shielding determination is based on the proton reference signal from liquid water in a spherical sample at a temperature of 34.7 °C, *σ_ref_*(^1^H_2_O_liq_._sph_) = 25.790(14) ppm [75]. Two experiments standardized this ^1^H signal: first, the simultaneous observation of the frequencies of an electronic and a proton transition in atomic hydrogen [76]; second, the simultaneous reading of ^1^H NMR signals from atomic hydrogen and pure water, both in the spherical samples [77]. The *σ_ref_*(^1^H) value can be directly applied to the scale of proton shielding, but the use of a spherical sample at elevated constant temperature is rather inconvenient in everyday NMR experimental practice. The ^1^H signal of H_2_O is extremely dependent on temperature. Figure 3 presents the positions of three isolated molecules (H_2_, H_2_O, and TMS) and the sample containing 1% of TMS in CDCl_3_ on the shielding scale relative to the *σ_ref_*(^1^H) reference signal. It permits the fast measurements of absolute shielding in ^1^H NMR spectra for gaseous and liquid compounds using ^1^H chemical shifts.

The actual *σ_ref_*(^1^H) parameter can also be used for shielding referencing other than proton nuclei as the universal reference standard of shielding, but it requires knowledge of the other nuclear magnetic dipole moments and the double-resonance experiments. There is also another possibility of transferring the shielding scale from one nucleus to another present in the same molecule, exploring the relaxation T_1_ time measurements in the gas phase. The latter method is described in detail by Jameson [75] and applied for the determination of the *σ_ref_*(^29^Si) in a gaseous mixture of SiH_4_ and SiF_4_ molecules [78], and the *σ_ref_*(^77^Se) parameter in H_2_Se and SeF_6_ molecules [32].

### 4.3. Helium-3 Atom as the Universal Shielding Reference

For many reasons, an isolated helium-3 atom is probably the best candidate for the universal reference standard of magnetic shielding in multinuclear NMR spectroscopy. First, the gas phase ^3^He NMR experiments delivered the resonance frequency of an isolated helium-3 atom at the stable external magnetic field [79]. Second, the ^3^He measurement is independent of temperature, and no rovibrational corrections are needed for further increase in accuracy. Third, the quantum chemical calculations deliver the most accurate value of the ^3^He atom, and this result can be accepted as the *σ_ref_*(^3^He) reference standard equal to 59.967 029(23) ppm [26].

The final step requires just the transfer of shielding from helium-3 to other magnetic nuclei experiments, and such a measurement can be taken using double resonance methods like those previously explored by McFarlane [33,34]. The comparison of Equation (8) for the ^3^He and another X nucleus in the same magnetic field (B) leads to Equation (10) and means the transfer of shielding from the helium-3 to the *X* nucleus. The experiment, according to Equation (10), requires only an NMR spectrometer and the nuclear dipole moments (*µ_He_* and *µ_X_*) [80,81,82]:

(10) $$\sigma_{X}=1-\frac{\nu_{X}}{\nu_{He}}\cdot \frac{\left|\mu_{He}\right|}{\left|\mu_{X}\right|}\cdot \frac{I_{X}}{I_{He}}\left(1-\sigma_{He}\right)$$

*I_X_* and *I_He_* are the nuclear spin numbers of the *X* and helium-3 nuclei, respectively. As seen in Equation (10), the knowledge of nuclear magnetic moments is crucial for the determination of shielding, and this problem is generally discussed in the next section.

## 5. Nuclear Magnetic Dipole Moments

As mentioned in the Introduction, the present state-of-the-art calculations of shielding are very powerful [12,13,14,15,16,17,18] and can often be used to improve our knowledge of nuclear magnetic shielding in molecules. The best theoretical results of magnetic shielding in molecules were also applied for the improvement of nuclear magnetic moments [83]. It was necessary because the existing results of the International Atomic Energy Agency (IAEA) at that time [84] were not reliable for all the heavier nuclei beyond hydrogen and helium-3. The determination of more accurate values of nuclear dipole moments was performed using the best available calculated results of shielding preferably in one molecule (*σ_X_* and *σ_Y_*) and the gas phase measurements of resonance frequencies for the same isolated molecule (*ν_Y_* and *ν_Y_*) where the index *X* refers to the reference nucleus, and *Y* is for the studied nucleus:

(11) $$\mu_{Y}=\frac{\nu_{Y}}{\nu_{X}}\cdot \frac{\left(1-\sigma_{X}\right)}{\left(1-\sigma_{Y}\right)}\cdot \frac{I_{Y}}{I_{X}}\mu_{X}$$

This way, the nuclear magnetic moment of the *X* reference nucleus was transferred to the other *Y* nucleus using NMR results for isolated small molecules and state-of-the-art shielding calculations performed for the same molecules. Protons and helium-3 were mostly used as the reference nuclei because their magnetic moments were repeatedly verified [26,80,81,84,85,86,87,88,89,90,91,92,93,94]. In some cases, the reference molecules could not contain protons, and other nuclei served as the reference *X* nuclei. The results obtained from the application of Equation (11) to isolated molecules are summarized in Table 2.

An interesting case of boron nuclei (^10^B and ^11^B) should be mentioned here, as the mixtures of ^3^He and BF_3_ gases were observed in the same gaseous samples [87]. The experiments have shown that such an approach with the direct comparison of shielding with helium-3 is precious, and later, it was also exploited for the measurements of magnetic moments of rare gases [93,94].

A bit less accurate results for heavier nuclei were available when the new experiments could not be performed in the gas phase. The magnetic dipole moments determined from NMR experiments in aqueous solutions and supported by the calculations of hydrated ions deliver fairly good results with the application of the same approach based on Equation (11). The improvements of *µ_X_* data were possible for numerous nuclei, mostly due to more accurate ab initio calculations: for ^6^Li, ^7^Li, ^23^Na, ^39^K, ^41^K, ^85^Rb, ^87^Rb, and ^133^Cs [95]; for ^9^Be, ^25^Mg, ^43^Ca, ^87^Sr, ^135^Ba, and ^137^Ba [96]; for ^27^Al, ^69^Ga, ^71^Ga, ^113^In, and ^115^In [97]; and ^45^Sc, ^89^Y, ^138^La and ^139^La nuclei [98]. All the above-cited data on nuclear magnetic dipole moments [83,86,87,88,89,90,91,92,93,94,95,96,97,98] were recognized by the International Nuclear Data Committee (INDC) as the standards and published in the IAEA documents [99].

Recently, the search for better and more accurate nuclear magnetic moments has continued. The most important study was published by Harding et al. [100]. The authors determined the nuclear magnetic moment with much higher accuracy for an unstable ^26^Na nucleus (1.1 s half-life time) using an improved version of the β-technique NMR combined with ab initio calculation of nuclear magnetic shielding performed for the stable ^23^Na reference. New studies and further possible improvements in the determination of magnetic moments for stable nuclei also appeared [101,102,103,104], and three review papers on the same subject were published [105,106,107].

## 6. Universal Approach to Shielding Measurements

**Figure 4:**
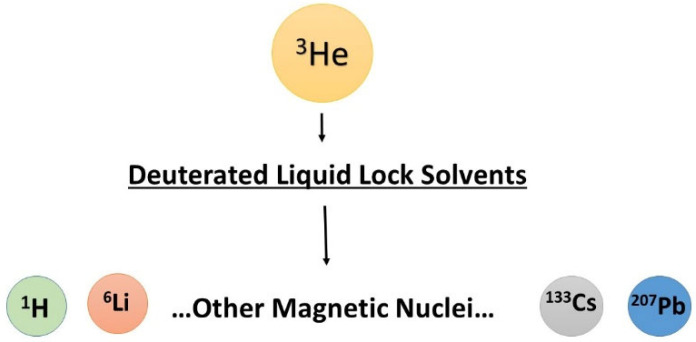
The absolute magnetic shielding known for an isolated helium-3 atom *σ_ref_*(^3^He) can be encoded into popular deuterated lock solvents [82,107]. Then, a standard NMR spectrometer is used as the double nuclear device, which permits the measurement of shielding for other magnetic nuclei. Such an experiment requires the exact reading of two resonance frequencies: *ν_X_* for the observed nuclei and ν_D_ for the deuterium lock solvent. Important, the nuclear magnetic dipole moments must be known with satisfactory accuracy for the above shielding measurements.

Nuclear magnetic shielding in molecules can be determined using a few different methods, as shown in Section 4. It is very helpful for the cross-checking of available experimental results and permits a better understanding of nuclear magnetic shielding. In our opinion, the application of Equation (10) is very promising because the number of reliable data for nuclear magnetic moments is quickly growing [83,86,87,88,89,90,91,92,93,94,95,96,97,98,99,100,101,102,103,104,105,106,107]. We also have an excellent reference standard of shielding, which is independent of temperature, the isolated helium-3 atom. However, the idea of the multinuclear experiments requires the simultaneous measurements of two different types of nuclei, the studied (*X*) and the reference nucleus (^3^He). Fortunately, it can be achieved using any standard NMR spectrometer with the deuterium lock system when first, the selected ^2^H NMR signal of lock solvent is calibrated by applying Equation (10), and then the deuterium lock solvent is used as the secondary reference of nuclear magnetic shielding [82]. Let us note that this way, all the shielding measurements remain referenced to an isolated helium-3 atom, *σ_ref_*(^3^He) = 59.9670 ppm [26]. In our opinion, an isolated helium-3 atom is the best choice for the primary reference standard of shielding in multinuclear NMR experiments. The above method is schematically presented in Figure 4.

Let us note that the new method of shielding measurements is well-calibrated with an isolated helium-3 atom and permits for using any standard NMR spectrometer in the double-resonance experiments according to Equation (12) where *ν_D_*, *µ_D_*_,_ and *σ_D_* are the frequency, ^2^H magnetic moment, and calibrated ^2^H shielding of liquid lock solvent, respectively; *I_D_* = 1.

(12) $$\sigma_{X}=1-\frac{\nu_{X}}{\nu_{D}}\cdot \frac{\left|\mu_{D}\right|}{\left|\mu_{X}\right|}\cdot I_{X}\cdot \left(1-\sigma_{D}\right)$$

Equation (12) can be generally used for all NMR samples if the lock solvent is separated from the observed substance. It only replaces the measurement of *δ_i_* in Equation (5) for the direct reading of shielding (*σ_i_* in Equation (5)). Both the parameters (*δ_i_* and *σ_i_*) are based on the same observation of experimental frequencies represented by *ν_i_* in Equation (6). In the present method of shielding measurements, nothing is modified inside the sample shielding (*σ_i_* in Equation (5)). Therefore, this method, described by Equation (12), can be safely used for the measurements of shielding in paramagnetic samples.

The *σ_D_* values of Equation (12) were calibrated for numerous signals from liquid lock solvents and published in original papers [82,107]. Table 3 below shows only the most popular solvents that are frequently used in NMR laboratories.

Equation (12) contains many constants that can be consolidated into one number for practical applications, for example, in the most popular ^1^H and ^13^C NMR experiments. The problem is then simplified to the reading of absolute frequencies (*ν_H_*) for protons or (*ν_C_*) for carbons-13 and simultaneously for deuterium nuclei (*ν_D_*) in lock solvent [82]:

(13) $$\sigma_{H}=1-\frac{\nu_{H}}{\nu_{D}}\cdot 0.153506104\cdot \left(1-\sigma_{D}\right)$$

(14) $$\sigma_{C}=1-\frac{\nu_{C}}{\nu_{D}}\cdot 0.610389782\cdot \left(1-\sigma_{D}\right)$$

**Figure 5:**
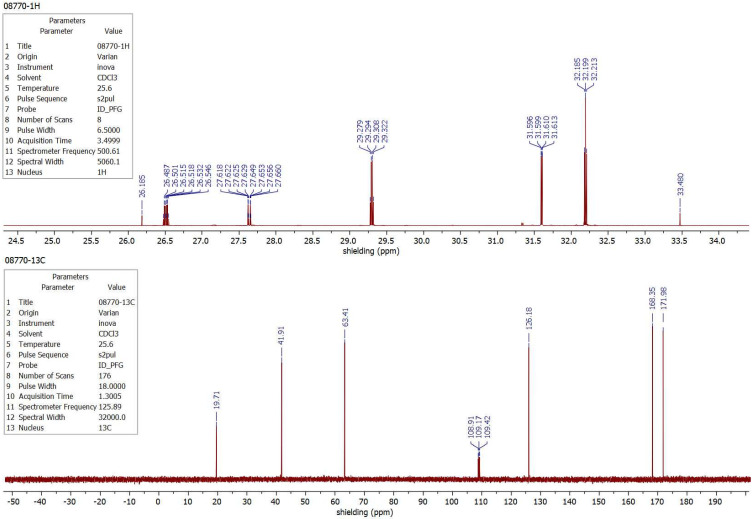
^1^H and ^13^C NMR spectra of liquid ethyl crotonate in CDCl_3_ on a 500 MHz Varian INOVA spectrometer. The ^2^H solvent signal of CDCl_3_ is used as the internal reference standard of nuclear magnetic shielding. The measurements contain all the components of shielding attributed to the particular nuclei in this sample.

At this point, everything is ready for the recording of multinuclear NMR spectra with the scale of magnetic shielding instead of chemical shifts. We are applying Equations (13) and (14) and the *σ_D_* parameters of Table 3 to get such a spectrum using a standard NMR spectrometer with a deuterium lock system. It is illustrated in Figure 5, where the ^13^C and ^1^H spectra of ethyl crotonate (CH_3_CH=CHCOOC_2_H_5_) dissolved in CDCl_3_ are shown as examples. It was possible to record them automatically due to a small modification of software in the computer of our 500 MHz VarianINOVA NMR spectrometer.

As shown, the measurements of nuclear magnetic shielding with the application of ^1^H and ^13^C NMR spectra are easy and comfortable for everyone. It does not require any special equipment and delivers valuable information on nuclear magnetic shielding. One feature of shielding measurements is especially interesting: the new method allows the measurement of the first-order isotope effects in shielding hydrogen isotopologues [108], which was impossible before [109]. All the isotope effects of hydrogen are illustrated by the original results presented in Table 4. Unexpectedly, the primary isotope effects in shielding ^0^Δ(^2/1^H) observed for H_2_, HD, and D_2_ molecules are stronger than the secondary isotopic effects ^1^Δ(^2/1^H) for the same molecules. It is an important measurement because standard NMR methods cannot be used to determine the primary isotopic effects in shielding. In this case, the direct measurements of ^1^H and ^2^H shielding were performed for the gaseous mixtures of hydrogen molecules with helium-3. All the frequency measurements were extrapolated to the zero-density limit, and Equation (10) was applied for the determination of all ^1^H and ^2^H shielding data in H_2_, HD, and D_2_ molecules [108].

The measurements of ^13^C magnetic shielding relative to *σ_ref_*(^3^He) were also extended on solid samples in MAS (Magic Angle Spinning) NMR spectra [111]. It was possible to use spherical samples of liquid TMS, a solution of 1% TMS in CDCl_3_, and solid fullerene C_60_ for the ^13^C shielding measurements in the standard NMR experiments, and the same samples were also observed by the MAS NMR method. Then, the ^13^C shielding values of popular MAS references like glycine, hexamethylbenzene, and adamantane were obtained by reading carbon-13 chemical shifts for solids [111].

Recently, the ^1^H, ^13^C, and ^14^N magnetic shielding parameters were measured for emodin and chuanxiongzine, plant products that have pharmacological properties and are frequently used in traditional Chinese medicine [112]. The direct measurements of ^1^H and ^13^C shielding were also applied in the studies of daidzein and puerarin which have natural anti-oxidant properties [113].

## 7. Conclusions

The origin of nuclear magnetic shielding in diamagnetic molecules is briefly discussed in the present review article. As shown, the important properties of molecules can be observed via chemical shifts in NMR spectra or can be calculated using advanced quantum chemical methods. The relations between shielding parameters and chemical shifts are rather complex because chemical shifts are separately defined for various magnetic nuclei by their reference standards. On the other hand, the measurements of shielding are badly needed for the direct comparison of experimental and calculated shielding values, also known as shielding constants. An isolated helium-3 atom is the natural choice for the universal reference standard of shielding. It can be easily applied in multinuclear NMR spectroscopy when its shielding is encoded into pure deuterated liquids that are used for the precise stabilization of the external magnetic field (lock system in NMR spectrometers). The method of shielding measurements is completed and can be applied to any liquid or gaseous NMR sample.

As shown in the previous section, the chemical shifts can be completely replaced in the future by the measurement of shielding parameters, and this alternative method of standardization of NMR spectra has numerous experimental advantages. The most important features of the new method are listed as follows. First, it unifies multinuclear methods into one NMR spectroscopy because the values of magnetic shielding have the same meaning independently of observed nuclei. Second, there is no need to use any additional reference standard if the NMR experiment is carried out with the calibrated ^2^H solvent, as shown in Table 3, because the same original reference standard of shielding is always preserved—an isolated helium-3 atom [82]. Third, the new method allows for the first time the measurement of the first-order isotope effects in shielding, as it has already been shown for hydrogen isotopologues [108]. Fourth, the measurements of ^13^C shielding relative to *σ_ref_*(^3^He) can be extended on solid samples in MAS NMR spectra, as shown in ref. [111]. Fifth, the measurements of shielding values are performed with the same precision as the standard determination of chemical shifts because they are based on the same reading of resonance frequencies, cf. Equation (6) vs. Equations (12)–(14). Last but not least, the determined shielding parameters can always be converted back into chemical shifts using Equation (5) if necessary and without the use of any additional reference standard. The simultaneous measurements of both the shielding parameters and appropriate chemical shifts are always available from the same single NMR experiment.

To summarize, the direct measurements of nuclear magnetic shielding are already available for selected light nuclei, and they are relatively easy to apply if the calibrated lock solvents are used as the secondary reference standards. It permits the use of every NMR spectrometer with a ^2^H lock system for required double-resonance experiments. We believe that progress in the knowledge of accurate nuclear moments will be growing fast, and the exact measurement of shielding will be available for more and more magnetic nuclei soon.

## Figures and Tables

**Table 1:** Spectral NMR parameters and recommended reference standards for selected magnetic nuclei.

NMR	Natural Abundance ^a^ [%]	*g_X_* Factor ^b,c^	Absolute Frequency ^a^ *Ξ_X_* [MHz]	Reference Shielding σ [ppm]	Recommended Reference Standard ^a^
^1^H	99.9885	5.585 694 70	100.000 000	33.480(5) ^d^	TMS in CDCl_3_
^2^H	0.0115	0.857 438 231	15.350 609	33.568(5) ^e^	TMS-d_12_ in CDCl_3_
^3^He	1.37 × 10^−4^	−4.255 250 62	76.179 437	59.967 ^f^	^3^He gas
^13^C	1.07	1.404 739	25.145 020	186.42(10) ^g^	TMS in CDCl_3_
^15^N	0.368	−0.566 141	10.136 767	−135.8 ^h^	CH_3_NO_2_ liquid
^17^O	0.038	−0.757 418 8	13.556 457	290.2 ^i^	D_2_O liquid
^19^F	100	5.256 68	94.094 011	188.7 ^j^	CCl_3_F liquid
^29^Si	4.6832	−1.110 104	19.867 187	379.0(20) ^g^	TMS in CDCl_3_
^31^P	100	2.261 85	40.480742	328.35 ^k^	H_3_PO_4_ 85% in H_2_O
^77^Se	7.63	1.06 1	19.071 513	2069 ^l^	(CH_3_)_2_Se liquid

^a^ Ref. [20]; ^b^ ref. [24]; ^c^ *g_X_* = *µ_X_*/(*I*_X_*µ_N_*), *µ_N_* = 5.050 783 53 × 10^−27^ J T^−1^; ^d^ ref. [25]; ^e^ estimated based on ^1^H results and the actual ^2^H resonance frequency, *Ξ_D_* (TMS-d_12_ in CDCl_3_); ^f^ ref. [26]; ^g^ ref. [25]; ^h^ ref. [27]; ^i^ ref. [28,29]; ^j^ ref. [30]; ^k^ ref. [31]; ^l^ ref. [32].

**Table 2:** Nuclear magnetic dipole moments are determined from the gas phase NMR measurements and quantum chemical calculations of shielding.

Applied NMR Methods	*µ_X_* Transfer	Observed Molecules	Magnetic Moments, *µ_X_*/*µ_N_*	More Details
^1^H and ^13^C	^1^H → ^13^C	^13^CH_4_	0.7023694(7)	Refs. [83,88]
^1^H and ^14^N	^1^H → ^14^N	^14^NH_3_	0.4035723(5)	Ref. [83]
^1^H and ^15^N	^1^H → ^15^N	^15^NH_3_	−0.283057(1)	Ref. [83]
^1^H and ^17^O	^1^H → ^17^O	H_2_^17^O	−1.893547(2)	Ref. [83]
^1^H and ^19^F	^1^H → ^19^F	CH_3_^19^F	2.62834(1)	Ref. [83]
^1^H and ^29^Si	^1^H → ^29^Si	^29^SiH_4_	−0.555052(3)	Ref. [86]
^1^H and ^31^P	^1^H → ^31^P	^31^PH_3_	1.130925(5)	Refs. [83,89]
^19^F and ^33^S	^19^F → ^33^S	^33^SF_6_	0.64325(2)	Ref. [83]
^1^H and ^35^Cl	^1^H → ^35^Cl	H^35^Cl	0.82170(1)	Ref. [90]
^1^H and ^37^Cl	^1^H → ^37^Cl	H^37^Cl	0.68398(1)	Ref. [90]
^1^H and ^73^Ge	^1^H → ^73^Ge	^73^GeH_4_	−0.87824(5)	Ref. [86]
^1^H and ^77^Se	^1^H → ^77^Se	H_2_^77^Se	0.53356(5)	Ref. [92]
^1^H and ^207^Pb	^1^H → ^207^Pb	^207^Pb(CH_3_)_4_	0.5906(4)	Ref. [91]
^3^He and ^10^B	^3^He → ^10^B	^10^BF_3_ + ^3^He	1.8004636(8)	Ref. [87]
^3^He and ^11^B	^3^He → ^11^B	^11^BF_3_ + ^3^He	2.688378(1)	Ref. [87]
^3^He and ^83^Kr	^3^He → ^83^Kr	^83^Kr + ^3^He	−0.970730(3)	Ref. [93]
^3^He and ^129^Xe	^3^He → ^129^Xe	^129^Xe + ^3^He	−0.77796(2)	Ref. [94]
^3^He and ^131^Xe	^3^He → ^131^Xe	^131^Xe + ^3^He	0.691845(7)	Ref. [94]

**Table 3:** Deuterium magnetic shielding in popular liquid lock solvents calibrated relative to an isolated ^3^He atom [82] *.

No	Lock Solvent	Observed ^2^H Signal	σ_D_ [ppm]
1	Cyclohexane-d_12_	-CD_2_-	31.834
2	Toluene-d_8_	-CD_3_	31.525
3	Acetonitrile-d_3_	-CD_3_	30.864
4	DMSO-d_6_	-CD_3_	30.574
5	Acetone-d_6_	-CD_3_	30.570
6	Methanol-d_4_	-CD_3_	29.593
7	Water-d_2_	-OD	28.837
8	Nitromethane-d_3_	-CD_3_	28.041
9	Benzene-d_6_	=CD-	26.441
10	Chloroform-d	-CD	26.389

* For liquids observed at the external parallel magnetic field (*B_ǁ_*) in 5 mm o.d. spinning NMR sample tubes at 300 K. More the σ_D_ parameters are available in ref. [107].

**Table 4:** The primary and secondary isotope effects from the direct measurements of shielding in H_2_, HD, and D_2_ isolated molecules [108] *.

Observed NMR Parameters	Primary Isotope Effects	Secondary Isotope Effects
Shielding in H_2_ and HD molecules	σ_0_(**H**_2_) = 26.293 ppm σ_0_(H**D**) = 26.239 ppm	σ_0_(**H**_2_) = 26.293 ppm σ_0_(**H**D) = 26.327 ppm
Isotope effects	**^0^** **Δ(^2/1^H) = −0.046 ppm**	**^1^** **Δ(^2/1^H) = −0.034 ppm**
Shielding in HD and D_2_ molecules	σ_0_(**H**D) = 26.327 ppm σ_0_(**D**_2_) = 26.388 ppm	σ_0_(H**D**) = 26.339 ppm σ_0_(**D**_2_) = 26.388 ppm
Isotope effects	**^0^** **Δ(^2/1^H) = −0.061 ppm**	**^1^** **Δ(^2/1^H) = −0.049 ppm**

* As defined in Ref. [110]: the ^0^Δ(^2/1^H) and ^1^Δ(^2/1^H) values mean the primary and secondary ^2/1^H isotope effects, respectively.
